# Quantitative Phosphoproteomics Identifies Myofibrillar Protein Phosphorylation Mediated by Pyruvate Kinase M2 in Beef

**DOI:** 10.3390/foods15071138

**Published:** 2026-03-26

**Authors:** Ying Xu, Xiangfei Liu, Chi Ren, Chengli Hou, Xin Li, Dequan Zhang

**Affiliations:** 1 College of Agriculture, Yanbian University, Yanji 133002, China; xuying114886@163.com; 2 Institute of Food Science and Technology, Chinese Academy of Agricultural Sciences/Key Laboratory of Agro-Products Quality & Safety in Harvest, Storage, Transportation, Management and Control, Ministry of Agriculture and Rural Affairs, Beijing 100193, China; liuxf1007@163.com (X.L.); candymce@163.com (C.R.); houchengli@caas.cn (C.H.)

**Keywords:** meat, pyruvate kinase M2, moonlighting function, myofibrillar protein, myosin regulatory light chain, phosphorylation

## Abstract

Pyruvate kinase M2 (PKM2) influences meat quality through glycolysis and also exhibits its moonlighting function as a protein kinase that catalyzes protein phosphorylation. However, it remains unclear whether PKM2 phosphorylates myofibrillar proteins, thereby affecting postmortem myofibrillar protein stability. This study investigates PKM2’s non-canonical kinase function using quantitative phosphoproteomics and an in vitro myofibrillar protein incubation model to identify its phosphorylation substrates and functional impacts. The quantitative phosphoproteomics identified 441 phosphoproteins, 881 phosphopeptides, and 1040 phosphorylation sites. Notably, the myosin regulatory light chain (MRLC) was identified as a likely candidate phosphorylation substrate of PKM2 in vitro. The interaction between PKM2 and MRLC was confirmed using co-immunoprecipitation (Co-IP) and Western blotting. Furthermore, MRLC phosphorylation by PKM2 significantly inhibited its degradation and enhanced its stability. This work establishes an in vitro biochemical framework for the moonlighting role of glycolytic enzymes, suggesting a potential mechanistic pathway that might influence myofibrillar protein stability during meat aging.

## 1. Introduction

Meat tenderness is a pivotal quality attribute that significantly influences consumer preferences and the economic sustainability of the meat industry [1]. This critical quality largely depends on the postmortem degradation of myofibrillar proteins, which constitute over half of total muscle proteins and form the structural backbone of muscle fibers [2]. Myosin, a key myofibrillar component, plays a central role in actomyosin interactions that influence muscle rigidity and tenderization. The dissociation of the actomyosin complex is widely recognized as a vital step in postmortem tenderization [3]. Therefore, any mechanism enhancing the stability of this complex, particularly by stabilizing key components like MRLC, was expected to hinder proteolysis and affect meat tenderness [4,5].

Our previous study demonstrated that protein phosphorylation modulates myofibrillar protein degradation in postmortem muscle, thereby influencing tenderness development [4]. Phosphorylation inhibits the activity of proteases like μ-calpain and alters protein susceptibility to degradation, with higher phosphorylation levels correlating to reduced tenderness across various species [6]. Recent phosphoproteomics analyses have identified over 100 phosphorylation sites on myofibrillar proteins, including desmin, actin, and myosin subunits, underscoring their dynamic regulation during rigor mortis and aging [7]. However, the kinases responsible for these modifications remain poorly understood, especially in relation to glycolytic enzymes that function as protein kinases under anaerobic postmortem conditions.

In postmortem meat, anaerobic glycolysis becomes the dominant metabolic pathway, profoundly affecting meat quality attributes such as pH decline, color stability, and ultimate tenderness. PKM2, a rate-limiting enzyme in this pathway, facilitates the conversion of phosphoenolpyruvate (PEP) into pyruvate, a process that also produces ATP. Elevated PKM2 expression has been linked to meat quality defects like pale, soft, exudative (PSE) conditions, which compromise tenderness and water holding capacity [8,9]. Beyond its traditional glycolytic function, PKM2 exhibits non-canonical roles, using PEP as a phosphate donor to phosphorylate target proteins without ATP involvement [10,11]. This moonlighting activity is particularly significant in energy-deprived environments where traditional ATP-dependent kinases are impaired [12,13]. Our previous work provided initial evidence of PKM2’s moonlighting function in meat science. We found that PKM2 enhanced the phosphorylation level of actin using an in vitro incubation model, supporting the hypothesis that PKM2 acts as a protein kinase in postmortem muscle to modulate meat quality [14]. However, direct evidence of this phosphorylation event within the muscle matrix is still lacking, and it remains unclear whether PKM2 can phosphorylate other myofibrillar protein substrates. These unresolved questions highlight a critical gap in our understanding of PKM2’s moonlighting functions in muscle.

This study investigated the substrates of PKM2’s moonlighting function by using quantitative phosphoproteomics in conjunction with an in vitro myofibrillar protein incubation model. We aimed to identify specific phosphorylation substrates and elucidate their functional impact on protein stability, thereby providing in vitro mechanistic insights into how PKM2 might interact with and stabilize myofibrillar proteins.

## 2. Materials and Methods

### 2.1. Muscle Sample Collection

The longissimus thoracis lumborum (LTL) muscles were collected from one side carcass of 15 Yanbian Yellow Cattle (12-month-old) at a local commercial slaughterhouse. The cattle were slaughtered according to standard commercial procedures. The meat samples were excised within 0.5 h postmortem. Immediately after excision, visible fat and connective tissues (fascia) were rapidly trimmed from the muscle surface. The samples were then frozen and stored at −80 °C until protein extraction.

### 2.2. Preparation of Dephosphorylated Myofibrillar Proteins

**Figure 1:**
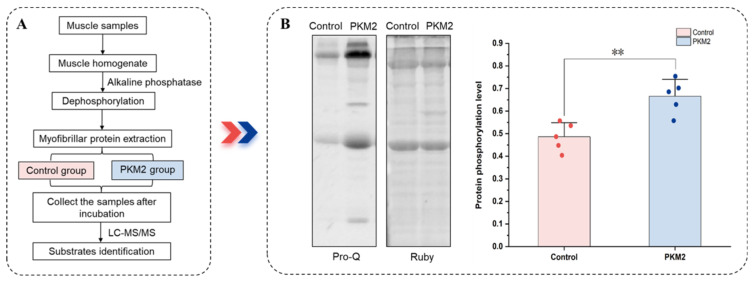
Quantitative phosphoproteomics approach to survey PKM2 Substrates. (**A**) The workflow for identifying the phosphorylation substrates of PKM2 using quantitative phosphoproteomics analysis. Myofibrillar proteins were extracted from the LTL muscles of 15 cattle. To minimize individual biological variability, protein extracts from every 5 randomly selected cattle were pooled to generate one biological replicate, resulting in a total of three independent biological replicates (*n* = 3) for the study. These three replicates were then divided into two experimental groups (Control and PKM2 group) and processed for TMT labeling and phosphoproteomics analysis. (**B**) The phosphorylation level of myofibrillar proteins in the in vitro reaction system. Data are presented as mean ± SD (*n* = 5). ** indicates *p* < 0.01.

Dephosphorylation of myofibrillar proteins was achieved with alkaline phosphatase to eliminate pre-existing phosphate groups, thus exposing additional residues for subsequent phosphorylation (Figure 1A). The muscle samples were first homogenized with buffer A (0.1 M KCl, 2 mM MgCl_2_, 2 mM EGTA, 0.02 M K_2_HPO_4_, pH 6.8) in a 1:10 proportion (*w*/*v*). The homogenate was first subjected to dephosphorylation by incubation with alkaline phosphatase (Sigma, St. Louis, MO, USA; 50 U/100 μg protein) at 30 °C for 1 h. Subsequently, myofibrillar proteins were isolated following the protocol from Kang et al. (2021), with minor adjustments [15]. After incubation, homogenates were centrifuged at 4 °C (2000× *g*, 15 min). The sediment was dispersed in buffer A containing 10% (*v*/*v*) Triton X-100 at a 1:8 ratio (*w*/*v*), followed by two rounds of centrifugation under the same conditions. The resulting precipitate, containing myofibrillar proteins, was dissolved in 0.1 M KCl.

### 2.3. In Vitro Phosphorylation Assay

The capacity of PKM2 to phosphorylate myofibrillar proteins was evaluated using an assay adapted from He et al. (2016) [10]. Myofibrillar proteins, previously dephosphorylated by alkaline phosphatase (Figure 1B), served as the substrate. The reaction was initiated by incubating these proteins in the buffer (100 mM KCl, 1 mM DTT, 50 mM MgCl_2_, 30 mM HEPES, 1 mM NaVO_4_, 5% glycerin, pH 7.5). The PKM2 treatment group was supplemented with PKM2 (0.1 μg/μL) and PEP (1 mM), whereas the control group contained no enzyme or PEP. The final concentration of myofibrillar proteins post-incubation was determined using the Pierce BCA Protein Assay kit (Thermo Fisher Scientific, Waltham, MA, USA). The final concentration of myofibrillar proteins in the PKM2 and control groups was 4 μg/μL. Both groups were incubated for 1 h at 4 °C, and samples were collected after incubation.

### 2.4. Co-Immunoprecipitation, SDS-PAGE and Western Blot

Co-IP of PKM2 was identified following the procedure based on the method of Ding et al. (2021) with minor adjustments [16]. According to instructions for the pre-treatment of protein A/G magnetic beads (HY-K0202, MedChemExpress (MCE), Shanghai, China). PKM2 antibody (A20991, ABclonal, Wuhan, China) of 2 μL was added to 80 μL of protein solution to form the protein complex and diluted with buffer (volume of 250 μL). The mixture was incubated overnight at 4 °C. After washing the magnetic beads, the protein complexes were mixed with the beads and incubated for 1 h. The beads were washed 3 times with buffer followed by one wash with ultrapure water. Subsequently, 100 μL of loading buffer was added to the centrifuge tube and incubated for 10 min. After removal of beads, eluate containing loading buffer was boiled in for 10 min water to obtain the SDS-PAGE sample for subsequent analysis of the interaction between PKM2 and MRLC. The sample of 5 μL was loaded onto SDS-PAGE gel (10% separating gel, 4% stacking gel). Then, the conditions for the Western blot transfer were 120 min at 300 mA. The primary antibodies were rabbit antiPKM2 (1:1000 for WB and 1:50 for IP, 4053S, CST, Danvers, MA, USA) and rabbit antimyosin regulatory light chain (1:1000 for WB, ab79935, Abcam, Cambridge, UK). The secondary antibody was HRP goat antirabbit IgG (1:1000, AS014, ABclonal, Wuhan, China).

Myofibrillar proteins were first resolved by SDS-PAGE using gels composed of the 10% resolving layer and the 4% stacking layer. Following electrophoresis, the gels underwent a sequential staining procedure according to the method of Chen et al. (2016) [17]. Specifically, phosphorylated proteins were visualized with Pro-Q Diamond (Invitrogen, Carlsbad, CA, USA), and subsequently, the overall protein profile was visualized with SYPRO Ruby (Invitrogen, Carlsbad, CA, USA).

Analysis of the MLRC’s phosphorylation level was performed using Zn^2+^-Phos-tag SDS-PAGE (Wako, Osaka, Japan). The Phos-tag gel electrophoresis was carried out (condition: 1 h, 30 mA). Then, protein transfer from the gel to a PVDF membrane was conducted at 250 mA over 2.5 h. The aforementioned primary (anti-myosin regulatory light chain) and secondary (HRP goat anti-rabbit IgG) antibodies were utilized for detection.

Myosin regulatory light chain degradation was examined via TGX stain-free gel (TGX Stain-Free FastCast^TM^ Acrylamide Kit, Bio-Rad, Hercules, CA, USA). Post-electrophoresis, gel was quantified for total proteins. The proteins were transferred onto a PVDF membrane at 100 V for 90 min. The membranes were then incubated with the antibodies as described above.

### 2.5. Analysis of Quantitative Phosphoproteomes

#### 2.5.1. Protein Preparation, Trypsin Digestion and Tandem Mass Tag (TMT) Labeling

Protein concentrations of in vitro-incubated samples (control and PKM2 groups) were assessed using the Pierce BCA Protein Assay Kit (Thermo Fisher Scientific, Waltham, MA, USA). Tryptic digestion of the proteins was performed according to the filter-aided sample preparation protocol. The resulting peptides were first purified using a C18 cartridge and then lyophilized to dryness. Finally, the dried peptide pellet was resuspended in 40 µL of 0.1% (*v*/*v*) formic acid. TMT labeling was performed on 100 µg of peptides from each sample using a TMT 10-plex kit (Thermo Fisher Scientific, Waltham, MA, USA), strictly adhering to the manufacturer’s protocol.

#### 2.5.2. Peptide Fractionation and Phosphopeptide Enrichment

The mixed, labeled peptide samples were loaded onto a C18 cartridge that had been previously conditioned with 0.1% TFA in acetonitrile. Unbound salts were then removed by washing the cartridge with ultrapure water. Bound peptides were then eluted in a stepwise manner with high-pH acetonitrile solutions. The fraction was vacuum-dried, resuspended in formic acid (concentration of 0.1%, 12 µL), and concentrations determined. Fractions were enriched for phosphopeptides using the High-Select^TM^ Fe-NTA Phosphopeptide Enrichment Kit (Thermo Fisher Scientific, Waltham, MA, USA). Enriched phosphopeptides were concentrated under vacuum and finally reconstituted in 20 µL of 0.1% formic acid for HPLC-MS/MS analysis.

#### 2.5.3. HPLC-MS/MS and Mass Data Analysis

Peptide samples were separated using a nanoflow HPLC system (Thermo Fisher Scientific, Waltham, MA, USA). Mobile phases consisted of (A) 0.1% (*v*/*v*) formic acid in water and (B) 0.1% (*v*/*v*) formic acid in acetonitrile/water (84:16, *v*/*v*). The analytical column (Thermo Scientific EASY-Spray column, 10 cm × 75 µm ID, packed with 3 µm C18-A2 particles) was pre-equilibrated with 95% of phase A. Samples were loaded onto a trapping column (Thermo Scientific Acclaim, Waltham, MA, USA PepMap100, 100 µm × 2 cm, nanoViper C18) at a flow rate of 300 nL/min and subsequently separated on the analytical column using a linear gradient (details provided in the gradient profile section, if applicable; otherwise, specify the gradient used).

Eluting peptides were identified using the Q-Exactive series mass spectrometer (Thermo Fisher Scientific, Waltham, MA, USA) under a data-dependent acquisition (DDA) scheme in positive ion mode. The settings for the full MS scan were as follows: mass range, m/z 300–1800; resolution, 70,000 (at m/z 200); AGC target, 1e6; and maximum IT, 50 ms. Dynamic exclusion was set to 60.0 s. For each full scan, the top 20 most intense precursor ions were selected for higher-energy collisional dissociation (HCD) fragmentation. MS/MS spectra were acquired with an isolation window of 2 m/z, a normalized collision energy (NCE) of 30 eV, a resolution of 17,500 (at m/z 200), an AGC target of 5 × 10^4^ (or specify if different; original text implies standard settings for MS2), and an underfill ratio threshold of 0.1%.

For phosphoproteomics data, a two-step normalization strategy involving sum normalization followed by median centering was applied. First, to eliminate variations in ionization efficiency among different proteins, the sum of intensities for each protein across all channels was calculated, and the intensity value in each individual channel was divided by this total sum. Subsequently, to correct for systematic technical biases between samples and ensure data comparability, the median intensity of all proteins within each channel was determined, and all values within that channel were divided by this median. The raw MS/MS files were imported into Proteome Discoverer 2.4 software (Thermo Fisher Scientific, Waltham, MA, USA) and queried against the Uniprot protein sequence database. The search parameters were configured with Trypsin/P as the digestion enzyme, permitting a maximum of two missed cleavages, and carbamidomethylation of cysteine (C) defined as a fixed modification. Oxidation and phosphorylation were specified as variable modifications. And false discovery rate (FDR) of protein, peptide and PSM was controlled at less than 1%.

### 2.6. Bioinformatics and Statistical Analysis

The data were presented as means ± standard deviation (SD). Differences in protein phosphorylation levels and degradation degrees between different treatment groups were analyzed using an independent-samples *t*-test with SPSS Statistics 19.0 (SPSS Inc., Chicago, IL, USA). The *p*-value less than 0.05 was considered statistically significant. For densitometric analysis, the intensity of protein bands was quantified using Quantity One 4.6.2 (Bio-Rad, Hercules, CA, USA).

Hierarchical clustering was conducted with the Cluster 3.0 (http://bonsai.hgc.jp/~mdehoon/software/cluster/software.htm, accessed on 1 March 2024), and the resulting heatmap was subsequently visualized using Java Treeview software (http://jtreeview.sourceforge.net, accessed on 1 March 2024). The differentially phosphorylated proteins were categorized into Gene Ontology (GO) terms, including biological process, cellular component, and molecular function, using the Blast2GO (https://www.ncbi.nlm.nih.gov/protein/, accessed on 1 March 2024). The differentially phosphorylated proteins were mapped to the Kyoto Encyclopedia of Genes and Genomes (KEGG) database (http://geneontology.org/) to identify their associated biological pathways. GO enrichment and KEGG pathway enrichment analyses were performed using Fisher’exact test. STRING database (https://string-db.org/) and IntAct molecular interaction database (http://www.ebi.ac.uk/intact/, accessed on 1 March 2024) were used for protein–protein interaction. Significance for enriched pathways and functional categories was determined based on an adjusted *p*-value of less than 0.05, which was calculated using the Benjamini–Hochberg procedure.

## 3. Results

### 3.1. Phosphorylation Levels of Myofibrillar Proteins in the In Vitro Incubation Model

An in vitro reaction system was developed to validate the role of PKM2 as protein kinase for phosphorylating myofibrillar proteins. In this system, dephosphorylated myofibrillar proteins were co-incubated with PKM2, alongside a parallel control group without the enzyme. The PKM2 group exhibited a significantly higher relative phosphorylation level than the control group (*p* < 0.05, Figure 1B). These results indicated that PKM2 effectively catalyzes the phosphorylation of myofibrillar proteins, affirming the successful establishment of the in vitro system, which is suitable for subsequent identification of its specific phosphorylation substrates.

### 3.2. Phosphoproteome Characterization of Myofibrillar Proteins

**Figure 2:**
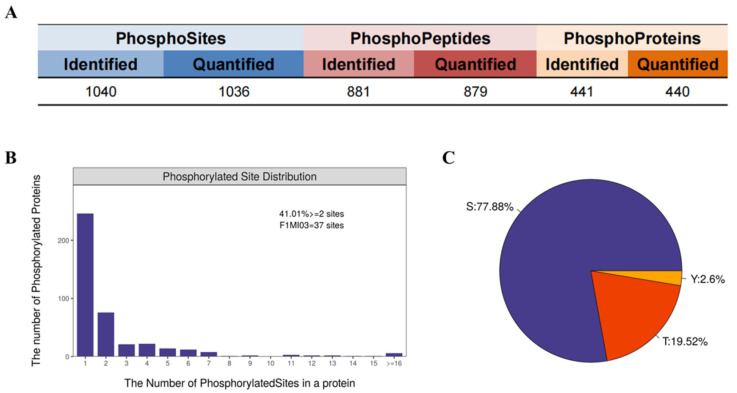
Characterization of phosphorylated proteomic data. (**A**) Identification and quantification results. (**B**) Distribution of the number of phosphorylation modification sites. (**C**) Ratio of the distribution of serine (S), threonine (T), and tyrosine (Y) phosphorylation modification sites.

A comprehensive bioinformatic analysis of mass spectrometry data was conducted to systematically characterize the features of PKM2-catalyzed phosphorylation in myofibrillar proteins from postmortem meat. The analysis identified 441 phosphoproteins, 881 phosphopeptides and 1040 phosphosites with high quantification completeness (Figure 2A). Further examination revealed that 41.01% of these proteins were multi-phosphorylated (i.e., at two or more sites). Phosphorylation predominantly occurred on serine (77.88%) and threonine (19.52%) residues (Figure 2B,C). These findings demonstrated that the phosphoproteomic dataset provides a solid foundation for subsequent screening of PKM2-specific substrates.

### 3.3. Comparative Analysis of Differentially Abundant Phosphopeptides

**Figure 3:**
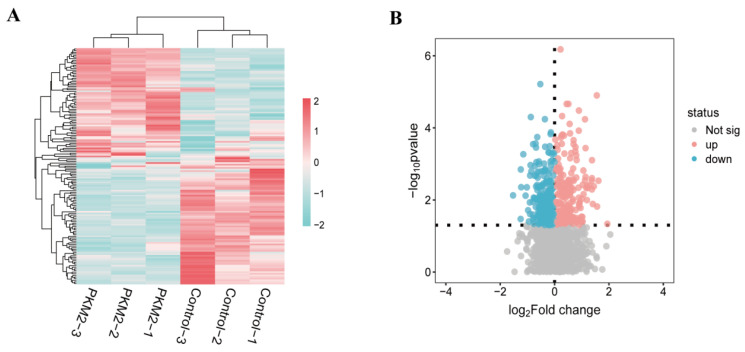
Analysis of differential abundant phosphopeptides (DAPs). (**A**) Hierarchical clustering analysis of the DAPs in Control/PKM2 group. (**B**) Volcano plot. Fold change set as >1.2 or <0.83 with *p* < 0.05. X-axis displays the log 2 (fold change), and Y-axis corresponds to the mean expression value of log 10 (*p*-value). Values for each phosphopeptide (row) of all analyzed samples (columns) are color coded based on the intensities of low (blue) and high (pink) Z-score normalized log 2 ratio (expression intensities). Grey indicates no quantitative information detected.

The hierarchical clustering heatmap revealed a distinct separation between control and PKM2 groups, indicating a significant difference in their phosphoproteomic profiles (Figure 3A). Using criteria of a fold change greater than 1.2 or less than 0.83 and a *p*-value less than 0.05, the volcano plot analysis identified 261 differentially abundant phosphopeptides. Among these, 139 phosphopeptides were significantly up-regulated, while 122 were significantly down-regulated in the PKM2 group (Figure 3B). These results demonstrated that PKM2 induces widespread and significant alterations in the phosphorylation status of myofibrillar proteins in postmortem muscle samples.

### 3.4. Annotation and Enrichment Analysis of Go Function and KEGG Pathway

**Figure 4:**
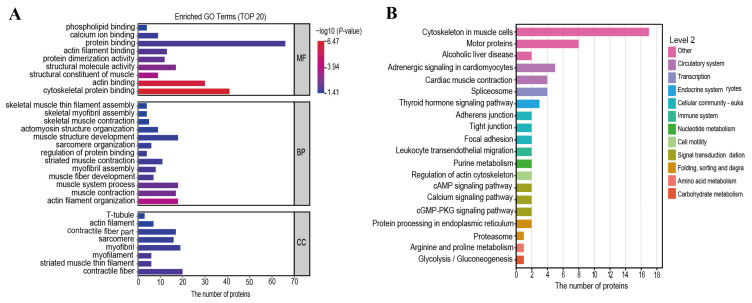
Go function and KEGG pathway annotation and enrichment analysis. (**A**) GO function enrichment histogram of proteins belonging to differentially expressed modified peptides. (**B**) KEGG pathway annotation and enrichment attribution histogram of proteins belonging to expression-modifying peptides.

GO functional and KEGG pathway enrichment analyses were performed to gain deeper insights into the biological functions of differentially phosphorylated proteins regulated by PKM2 during postmortem tenderization. GO functional enrichment analysis identified core functional categories of these proteins (Figure 4A). Results revealed significant enrichment of differentially phosphorylated proteins across biological process (BP), cellular component (CC), and molecular function (MF). At the BP level, these proteins were significantly enriched in muscle contraction, actomyosin structure organization, and muscle system process. At the CC level, they localized primarily to contractile units, including T-tubule, actin filament, contractile fiber part, sarcomere, myofilament, striated muscle thin filament, and contractile fiber. The enriched MF terms focused on binding functions, such as protein binding, cytoskeletal protein binding, actin binding, and serving as structural constituents of muscle. Furthermore, the hierarchical and subordinate relationships among these key GO terms were illustrated (Figure 4A), which reaffirmed the central role of the differentially phosphorylated proteins in muscle structure and contractile activity.

Differentially phosphorylated proteins were analyzed using KEGG pathway enrichment to elucidate their associated signaling pathways (Figure 4B). The enriched KEGG pathways were primarily related to muscle contraction (cytoskeleton in muscle cells, motor proteins, and regulation of actin cytoskeleton), adhesion (adhesions junction, tight junction and focal adhesion) and metabolism (amino acid metabolism and glycolysis/gluconeogenesis). Additionally, glycolysis is the core metabolic process governing ATP production and pH decline in postmortem muscle, while pathways such as focal adhesion and actin cytoskeleton regulation are directly linked to the structural integrity and stability of the myofibril. These findings provided compelling evidence that PKM2-mediated phosphorylation may influence the energy status of muscle while also alter the phosphorylation state of myofibrillar proteins. The interplay between these pathways likely constitutes the underlying molecular mechanism by which PKM2 regulates postmortem actomyosin dissociation and meat tenderness ultimately.

### 3.5. Constructing of Protein–Protein Interaction Network

**Figure 5:**
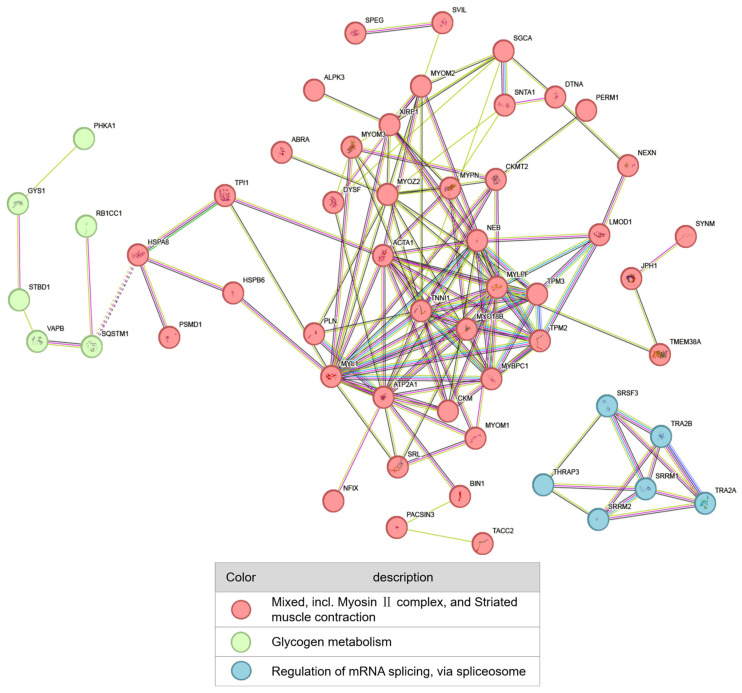
Protein–protein interaction networks of differentially phosphorylated proteins.

A protein–protein interaction (PPI) network was constructed using the identified differentially phosphorylated proteins to explore phosphorylated targets of PKM2 and their functional networks in postmortem muscle (Figure 5). The PPI network was distinctly divided into three major functional modules: myofibrillar structure (red), glycogen metabolism (green), and RNA splicing (blue). The largest module, related to the myofibrillar structure, comprised core structural proteins such as MYL1 and ACTA1. This result indicated that postmortem muscle maturation involves coordinated phosphorylation events across multiple levels, including structural, metabolic, and regulatory processes. The differentially phosphorylated proteins depicted constitute a pool of potential substrates regulated by PKM2, providing key candidates for subsequent validation of its specific targets.

### 3.6. Validation of PKM2’s Effect on the Phosphorylation and Degradation of MRLC

**Figure 6:**
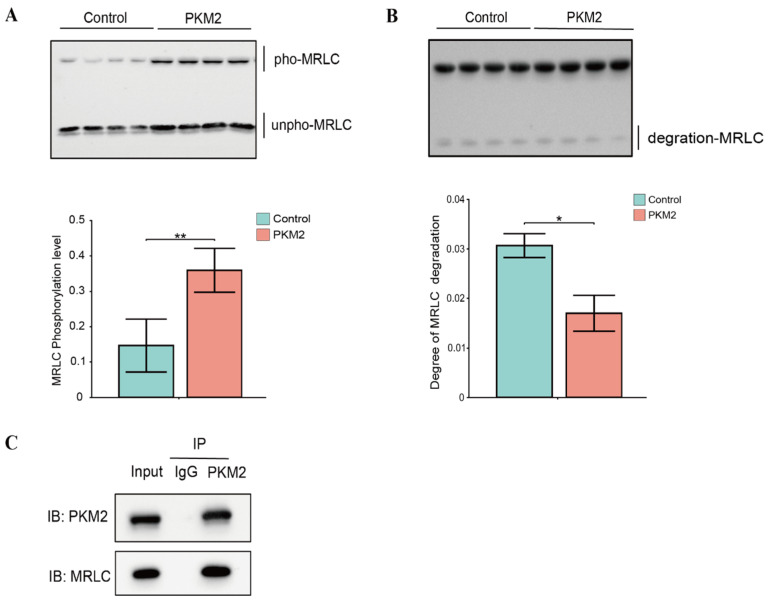
Validation of PKM2-mediated phosphorylation and stabilization of MRLC. (**A**) Western blot and corresponding quantification of myosin regulatory light chain (MRLC) phosphorylation in the absence (Control group) and presence (PKM2 group) of PKM2. pho-MRLC: phosphorylated MRLC; unpho-MRLC: unphosphorylated MRLC. (**B**) Western blot analysis showing that PKM2 inhibited the degradation of MRLC. The bar chart shows the quantification of MRLC degradation fragments. (**C**) Co-immunoprecipitation (Co-IP) assay confirming the direct interaction between PKM2 and MRLC. Lysates were immunoprecipitated with an anti-PKM2 antibody and immunoblotted (IB) for MRLC. Data are expressed as mean ± SD from four independent experimental replicates (*n* = 4). * indicates *p* < 0.05, ** indicates *p* < 0.01.

Our quantitative phosphoproteomics analysis identified MRLC as a potential substrate of PKM2 (Table S1). To validate this finding, we employed a series of biochemical assays to confirm this interaction and investigated the functional impact of PKM2-mediated phosphorylation on MRLC. The Co-IP analysis revealed an interaction between PKM2 and MRLC (Figure 6C). An in vitro kinase assay demonstrated that the addition of PKM2 significantly increased the phosphorylation level of MRLC (*p* < 0.05, Figure 6A). Interestingly, PKM2 presence markedly inhibited the degradation of MRLC (*p* < 0.05, Figure 6B). These results indicated that MRLC was a candidate substrate of PKM2. The addition of PKM2 likely catalyzes its phosphorylation, thereby inhibiting its degradation and enhancing its stability.

## 4. Discussion

This study provides the first direct evidence that PKM2, a key glycolytic enzyme, moonlights as a protein kinase that phosphorylates myofibrillar proteins in the postmortem muscle environment. MRLC was identified as a principal substrate of PKM2. This discovery offers crucial mechanistic insight, as the upstream kinases involved in post-translational modifications, particularly protein phosphorylation, are a central yet incompletely understood aspect of meat quality development. It is increasingly recognized that the phosphorylation status of structural proteins influences their susceptibility to enzymatic degradation, thereby affecting the rate and extent of tenderization [4]. Specifically, the phosphorylation of MRLC critically affects actomyosin dissociation, a prerequisite for the subsequent proteolytic breakdown of myofibrils that underlies tenderness improvement postmortem [5,18]. While previous research has documented these phosphorylation events, the upstream kinases responsible, especially those active under the unique anaerobic and energy-depleted conditions of postmortem muscle, have remained largely elusive. Our findings offer insights into a potential regulatory axis where PKM2, a key metabolic enzyme, also acts on the structural integrity of the contractile apparatus, suggesting a new molecular framework for understanding the intricate regulation of postmortem muscle.

The quantitative phosphoproteomics analysis, which identified 881 differentially abundant phosphopeptides following incubation with PKM2, substantiates the enzyme’s potent and widespread kinase activity. The functional enrichment of these target proteins in muscle contraction, myofibril structure and actin binding compellingly supports our core hypothesis that PKM2’s kinase activity directly targets the muscle’s contractile machinery. The significance of this finding is particularly pronounced in the biochemical context of postmortem muscle, an environment where rapid ATP depletion and the dominance of anaerobic glycolysis necessitate alternative signaling pathways [19,20,21]. PKM2’s demonstrated ability to utilize PEP as an alternative phosphate donor confers an ATP-independent kinase activity, positioning it as a critical “moonlighting” kinase [12]. It remains active precisely when canonical, ATP-dependent kinases are progressively inactivated. Based on these in vitro findings, we hypothesize that the moonlighting kinase activity of PKM2 might represent an intricate biochemical cascade that regulates the stability of myofibrillar proteins in postmortem muscle. Furthermore, the significant enrichment of the Glycolysis/Gluconeogenesis pathway leads us to speculate about a complex autoregulatory loop. In this proposed loop, PKM2 might modulate the phosphorylation status of other glycolytic enzymes, thereby potentially influencing energy metabolism and the rate of pH decline [12,20].

The PPI network provided a systems-level view of PKM2’s impact, clearly segregating its substrates into distinct functional modules. Beyond MRLC, our quantitative phosphoproteomics dataset provides a comprehensive landscape of potential PKM2 substrates, warranting broader interpretation. The preeminence of the myofibrillar protein cluster, which comprises skeletal muscle actin (ACTA1), other MRLC isoforms (e.g., MYL1), and other structural components, compellingly identifies the contractile apparatus as the primary target of this non-canonical activity. Specifically, actin forms the backbone of thin filaments, and its phosphorylation has been reported to alter the spatial conformation of the actomyosin complex, potentially affecting meat toughness [22]. This network model corroborates findings from recent large-scale phosphoproteomic studies in postmortem muscle, which have cataloged extensive phosphorylation of myofibrillar proteins but were unable to pinpoint the responsible upstream kinases [14]. Our study fills this critical knowledge gap by experimentally demonstrating that PKM2 is a key upstream regulator. Furthermore, the identification of smaller, yet significant, clusters related to glycogen metabolism and RNA splicing implies that PKM2’s regulatory scope is broader than initially anticipated. Supported by the significant enrichment of the Glycolysis/Gluconeogenesis pathway, this strongly suggests that PKM2’s moonlighting kinase activity extends beyond structural proteins to include metabolic enzymes. This implies a potential autoregulatory feedback loop within postmortem muscle, where PKM2 might phosphorylate upstream or downstream glycolytic enzymes, thereby collectively fine-tuning the rate of postmortem glycolysis. Exploring these additional structural and metabolic targets will be crucial for fully mapping the PKM2-driven biochemical network.

The co-IP experiment confirmed a direct physical interaction between PKM2 and MRLC, while the subsequent kinase assay unequivocally demonstrated that PKM2-catalyzed phosphorylation of MRLC significantly inhibited its degradation. This result delineates a precise molecular mechanism linking PKM2 to the structural stability of myofibrillar proteins. MRLC phosphorylation is a well-established regulator of actomyosin dynamics, for instance, phosphorylation by myosin light chain kinase is typically associated with promoting muscle contraction [5,23,24]. Cao et al. (2021) showed that MRLC phosphorylation at Ser17 regulates actomyosin dissociation [5]. This study introduced PKM2 as a novel player in the post-translational modification of contractile proteins, and demonstrated that its phosphorylation of MRLC serves a distinct function by enhancing the protein’s stability. The tenderization process is fundamentally reliant on the proteolytic degradation of key myofibrillar proteins, such as MRLC, by endogenous proteases like calpains and caspases, leading to the weakening of the myofibril structure [6,25,26]. Our in vitro assay provides direct preliminary biochemical evidence that PKM2-catalyzed phosphorylation renders MRLC highly resistant to spontaneous degradation. Mechanistically, the addition of a bulky, negatively charged phosphate group by PKM2 can induce local conformational changes and create significant steric hindrance [27,28]. This structural alteration likely obscures the specific cleavage sites required by endogenous proteases. Previous studies have robustly demonstrated that MRLC phosphorylation regulates actomyosin dissociation and subsequent meat tenderness [5]. Drawing on this established downstream relationship, we hypothesize that by phosphorylating MRLC and sterically hindering its proteolysis in vitro, PKM2 represents a biologically plausible upstream mechanism that might “lock” the actomyosin complex. While we speculate that this could delay the tenderization process and contribute to the maintenance of muscle structure, it must be emphasized that its actual physiological impact on postmortem actomyosin dissociation remains a theoretical hypothesis requiring direct in vivo testing. The discovery of PKM2’s moonlighting function extends its known roles in cancer biology into the realm of food science, providing a new paradigm for meat quality regulation [11,29].

However, it is crucial to acknowledge the limitations of the current in vitro assay system. Although these data provide a strong biochemical proof-of-concept, extrapolating these specific in vitro findings to the highly complex and dynamic matrix of intact postmortem muscle should be interpreted with caution. While the robust in vitro conditions used in this study successfully captured the maximum kinase potential of PKM2 and facilitated reliable substrate identification, we acknowledge certain limitations. Specifically, the supra-physiological concentrations of PKM2 and PEP were deliberately employed to maximize the phosphoproteomics signal. Our in vitro model provides robust evidence for the PKM2-MRLC regulatory axis, and the next crucial step is to validate these findings in a postmortem context. Future research should focus on quantifying the dynamics of MRLC phosphorylation at PKM2-specific sites during postmortem storage. Furthermore, harnessing this newly discovered mechanism holds considerable potential for applied meat science. If validated in situ, strategies to modulate PKM2’s kinase activity could offer a novel biochemical perspective for understanding postmortem myofibrillar protein dynamics.

## 5. Conclusions

This study identified a suite of potential myofibrillar protein substrates for the kinase activity of PKM2 using quantitative phosphoproteomics analysis, unveiling a molecular regulatory network that influences myofibrillar protein integrity. Our findings fundamentally advance the understanding of the moonlighting function of the glycolytic enzyme PKM2 as a protein kinase, identifying MRLC as a key candidate substrate in vitro. This phosphorylation event enhanced the stability of MRLC and inhibited its subsequent degradation. This discovery establishes a previously unrecognized link between postmortem muscle proteins status and myofibrillar protein stability. It provides a new in vitro biochemical framework for understanding the moonlighting functions of key glycolytic enzymes and suggests a potential mechanistic pathway that could relate to the structural changes in muscle proteins during postmortem aging upon further in situ validation.

## Data Availability

The mass spectrometry proteomics data have been deposited to the ProteomeXchange Consortium (https://proteomecentral.proteomexchange.org, accessed on 20 February 2026) via the iProX partner repository [30,31] with the dataset identifier PXD075382. The original contributions presented in the study are included in the article/Supplementary Material, further inquiries can be directed to the corresponding authors.

## Supplementary Materials

The following supporting information can be downloaded at: https://www.mdpi.com/article/10.3390/foods15071138/s1, Table S1: Analysis of significant differences in phosphopeptide.

## Abbreviations

The following abbreviations are used in this manuscript:

PKM2	Pyruvate kinase M2
MRLC	Myosin regulatory light chain
Co-IP	Co-immunoprecipitation
